# PROTOTYPE OF A VIRTUAL REALITY APPROACH TO INTEGRATE MENTAL HEALTH ACROSS GERONTOLOGICAL ENVIRONMENTS

**DOI:** 10.1093/geroni/igz038.1893

**Published:** 2019-11-08

**Authors:** Daniel Velez Ortiz

**Affiliations:** Michigan State University, East Lansing, Michigan, United States

## Abstract

Background: Puerto Ricans have the highest likelihood of psychiatric disorders among Latinos. This study developed and evaluated a prototype depression literacy curriculum; culturally grounded with perspectives and narratives of Puerto Rican older adults. The way a person determines need for services and decides to seek help has been found to be influenced by their perceptions of services and providers. McGuire (1989) presents the Communication Persuasion Model (CPM) that takes into account how persuasive communication changes attitudes and behaviors of consumers. Using the CPM as a theoretical foundation, this study presented a culturally grounded story through a Virtual Reality (VR) platform. Methods: A script was developed based on narratives of Puerto Rican older adults about depression. Filmed in 360° format and enhanced with supporting imagery, participants were presented two versions of the video, one with a VR headset and the other with a smartphone. Two focus group interviews were conducted with community-dwelling Puerto Rican older adults (n=14) in Orlando, FL. Results: Participants preferred the VR headset and found it was beneficial to educate about depression because it felt more immersive and encouraged an environment conducive to identifying their own experiences about depression. They noted that presenting the material with a case narrative was more culturally sensitive for the population. All participants needed minor assistance with operating technology. Conclusions and Implications: A narrative approach to depression literacy may be effective in personalizing messages. Assisted VR technology with supporting imagery may be efficacious and standardize positive messages to underrepresented and low resource populations.

